# Amusia Results in Abnormal Brain Activity following Inappropriate Intonation during Speech Comprehension

**DOI:** 10.1371/journal.pone.0041411

**Published:** 2012-07-27

**Authors:** Cunmei Jiang, Jeff P. Hamm, Vanessa K. Lim, Ian J. Kirk, Xuhai Chen, Yufang Yang

**Affiliations:** 1 Music College, Shanghai Normal University, Shanghai, China; 2 Institute of Psychology, Chinese Academy of Science, Beijing, China; 3 Research Centre for Cognitive Neuroscience, The University of Auckland, Auckland, New Zealand; 4 School of Psychology, Shaanxi Normal University, Xian, China; Goldsmiths, University of London, United Kingdom

## Abstract

Pitch processing is a critical ability on which humans’ tonal musical experience depends, and which is also of paramount importance for decoding prosody in speech. Congenital amusia refers to deficits in the ability to properly process musical pitch, and recent evidence has suggested that this musical pitch disorder may impact upon the processing of speech sounds. Here we present the first electrophysiological evidence demonstrating that individuals with amusia who speak Mandarin Chinese are impaired in classifying prosody as appropriate or inappropriate during a speech comprehension task. When presented with inappropriate prosody stimuli, control participants elicited a larger P600 and smaller N100 relative to the appropriate condition. In contrast, amusics did not show significant differences between the appropriate and inappropriate conditions in either the N100 or the P600 component. This provides further evidence that the pitch perception deficits associated with amusia may also affect intonation processing during speech comprehension in those who speak a tonal language such as Mandarin, and suggests music and language share some cognitive and neural resources.

## Introduction

It has been suggested that humans are predisposed to process melodies in a holistic manner [1]. Consistent with this is the finding that before the age of 1 year infants can perceive and recognize musical patterns of pitch [2]. However, 4% of the general population in the United Kingdom [3], and 3.4% in China [4] have problems in the perception of musical pitch. This pitch related disorder is known as congenital amusia (amusia hereafter) [5]. Individuals with amusia have difficulties in fine-grained pitch discrimination [6]–[9], pitch contour discriminations [6], [10], anomalous pitch detection, dissonance-pleasantness judgments, and tune recognition from songs [11]. They may also show a mismatch between pitch perception and production abilities [12]. Recent studies suggest that the pitch related deficits are associated with impairments of pitch memory [13]–[15].

Amusia has been associated with a decrease in white matter and thicker cortex in the right inferior frontal gyrus [16]–[17]. It has also been reported that amusics exhibit reduced gray matter volume in the left inferior frontal gyrus [18], an abnormally reduced arcuate fasciculus in the right hemisphere [19], and reduced connectivity between the right inferior gyrus and corresponding auditory cortex [20]. Along with these structural changes, brain functional changes have been shown using electroencephalography (EEG). It has been demonstrated, for example, that relative to controls individuals with amusia do not as reliably elicit brain activity in response to pitch changes smaller than one semitone. In addition, they ‘overreact’ to large pitch changes by eliciting an N200 that is not found in the controls, and produce a larger P300 effect [21]. Within a musical context, although a quarter-tone pitch difference elicited the N200 effect it did not elicit an expected P600 effect in amusics. This pattern of ERP effects was suggested to indicate that the acoustic information does not appear to integrate into a conscious percept in amusia [22].

Proponents of the resource-sharing framework argue that music and language share neural resources, although each has a specialized representation [23]–[25]. In contrast, proponents of the modularity view consider that music and language each have their own module or domain specificity [26]–[28]. Pitch, which is fundamental to the melody in music, is also important with respect to prosody in speech [29]. Whether or not the musical pitch deficits in amusia extend to prosodic processing in speech is hotly debated.

Although amusia is thought of as a music-specific deficit involving pitch detection and identification [9], [11], some studies suggest that the deficit in pitch processing may extend to pitch discrimination in spoken syllables [30], lexical tones [31], and affects the intonation perception of prosody [32]–[35]. Support for this latter suggestion has been shown in that amusics show impaired processing of emotional prosody [36]. These speech related pitch perception deficits also occur for amusic speakers of tonal languages. It has been demonstrated that Mandarin amusics have impaired lexical tone identification [4] and discrimination [37], and lack categorical perception of Mandarin tones [38]. Although there are slight differences in the processing of intonation for natural speech between [10] and [37], differences which are attributed to the aid of the non-pitch-based cues of duration and intensity in speech perception [37], Mandarin amusics have shown problems in the processing of non-linguistic analogues derived from statements and questions [10], [37], as is found with amusic speakers of non-tonal languages [11], [33]–[34]. This may be related to the amusics’ impairments in identifying the direction of a change in pitch for non-linguistic analogues [39].

It has been reported that prosodic perception aids speech comprehension for speakers of both tonal and non-tonal languages [40]–[41]. Although amusics have impaired intonation perception in prosody [10], [32]–[35] and emotional prosody [36], none of the individuals with amusia in the previous studies reported having deficits with everyday speech comprehension [8], [10], [37]–[39]. If this lack of day to day deficits is due to normal speech containing additional non-pitch-based cues [37], then laboratory experiments must employ techniques that are capable of detecting subtle deficits.

Event-related potentials (ERPs) can provide information on the neuronal activity related to speech comprehension with millisecond accuracy. Although ERPs have been employed to examine the neural dynamics of musical pitch processing in amusia [21]–[22], the neural bases of the speech related pitch deficits in amusia remain uncertain. On the other hand, emerging evidence on domain-transfer effects suggests that tonal language experience may facilitate processing in both music (e. g. [42]–[43]) and speech (e. g. [44]–[45]), however, previous behavioral studies have found that Mandarin language experience does not compensate for the pitch deficits associated with amusia [4], [8], [10], [37]–[39]. In this case, to examine the neural bases of intonation processing during speech comprehension in Mandarin speaking amusics, the current study recorded brain activities during a task that relied on speech related pitch sensitivity. This may shed light on the nature of the pitch deficits in amusia, and provide additional evidence towards comparisons between music and language.

ERP effects that are known to be linked to the semantic aspects of language include the N400 effect [46]. The N400 effect manifests as a relatively more negative going wave over parietal electrodes for words that do not conform to semantic expectations relative to words that do [46]–[47], even when the words are presented in isolation and semantically primed by pictures [48]–[49] or gestures [50]. In addition to the above semantic aspects of language processing, syntactical processing is also indexed by ERP effects. The P600 effect is commonly associated with the processing of syntactic violation with grammatical errors [51]–[53]. However, the P600 effect is also elicited in the absence of grammatical errors by semantic attraction [54], temporary misanalysis (garden paths) [51], [55], and violations of constraints on long-distance dependencies [56]. Mismatches between syntax and prosody can also induce the garden path effects which are indexed by the P600 effect [57]–[58]. The P600 family of effects includes a frontally distributed effect, which appears to reflect a revision process [59], and a more posteriorly distributed effect, which appears to indicate syntactic processing difficulty in repair and revision processes [51].

It is the finding that the P600 effect is generated by syntax-prosody mismatch [57]–[58] that is of most interest to the current study. Interaction of prosodic and syntactic processes in speech comprehension has been reported not only in Mandarin Chinese [60]–[61], but also in western languages, such as English, Dutch, and French [62]–[65]. When prosody is consistent with syntax, it can facilitate syntactic parsing (e. g., [66]–[67]), whereas inconsistencies between prosodic and syntactic structure induce processing difficulties (e. g., [68]–[69]). It has been suggested that the P600 effect for inappropriate prosody is not induced because this is a rare or infrequent occurrence, but because of the inadequate or inappropriate aspect of the intonation [70].

To examine whether or not Mandarin speaking amusics show abnormal brain activity to inappropriate prosody during speech comprehension, the current study manipulated intonation during judgments of semantic acceptability in short speech discourses. Appropriate intonation signifies prosody-syntax match, while inappropriate intonation results in a mismatch between prosody and syntax. If the pitch deficits associated with amusia are not music specific, but also have a negative impact upon speech comprehension, then the brain activities of amusics in response to inappropriate intonation should differ from those of normal controls. More specifically, if amusics are impaired in their detecting of prosody, then use of inappropriate intonation should be less detectable. In this case, the utterance is more likely to be interpreted as directed by the semantics [59], thus resulting in the impression that it seems correct. In contrast, the controls should show a P600 effect [70] and improved detection performance relative to the amusic group.

## Methods

### Participants

Eleven amusics and eleven controls participated in the current study. Among these participants, half of them (4 amusics and 7 controls) had participated in our previous studies [8], [10] with the remainders being new volunteers who were recruited in the same way as those amusic participants in our previous studies [8], [10]. All were undergraduates or postgraduates with Mandarin Chinese as their first language and were recruited by advertisements posted on the bulletin board system of universities in Beijing. None had received extra curriculum music training. None reported a history of neurological, psychiatric diseases, hearing difficulties, or difficulty in speech communication. Hand dominance was assessed by the Edinburgh Handedness Inventory [71]. They were divided into 18 right-handers, and 4 left-handers (2 amusic and 2 control participants, respectively).

The musical abilities of all the participants were tested by the Montreal Battery of Evaluation of Amusia including the scale, contour, interval, rhythm, meter, and memory subtests (MBEA) [72]. Table 1 presents the participants’ characteristics, global (overall average), and melodic (average of the scale, contour, and interval subtests) scores of the MBEA. Ethical approval was attained from the Institute of Psychology, Chinese Academy of Sciences, and written informed consent was obtained from all of the participants.

**Table 1:** Participants’ characteristics and mean scores from the MBEA for each group.

	Amusic (n = 11)	Control(n = 11)	t-test
Mean age (SD)	23 (2.7)	24 (1.4)	NS
Sex	8F, 3 M	7F, 4 M	
Years education (SD)	16 (2.3)	17 (1.5)	NS
Global score of MBEA (SD)	19 (2.5)	27 (1.2)	*p*<0.001
Melodic score of MBEA (SD)	18 (2.3)	27 (1.3)	*p*<0.001

Note: F = female; M = male.

### Materials

In Mandarin, focus (discourse/pragmatic motivated emphasis) plays a critical role in distinguishing between a question and a statement [73]. For Mandarin sentences with final focus, the difference between a statement and a question occurs mainly in the final words [73]–[75].

One hundred thirty-six short discourses including question-answer pairs were spoken by a female native speaker of Mandarin Chinese. Each answer sentence contained two parts. The first part was to answer directly the question with yes or no, and the second was a two clause sentence where the first clause explained the reason and the second was either a relevant statement or question. For example,

Q: 今天飞机起飞吗?　 (Is the plane taking off today?)

A: 不。雾气这么大,飞机停飞。/? (No. The fog is too thick, and the plane is grounded./?)

The end of the discourse is a verb-object construction with final focus, the verb and object consisting of one syllable each. This construction is infrequent (mean ± SD  = 95.27±54.66, per million) in Mandarin Chinese [76]. Each of the 136 short discourses was spoken twice, once with the final syllables spoken as a question and once as a statement. As a result, each discourse has two spoken versions: one with an appropriate intonation and the other one with an inappropriate intonation. More specifically, if from a semantic perspective a given discourse should end with a question and it is actually spoken as a question then this is an appropriate intonation. However, if this discourse was spoken as a statement then this would be an inappropriate intonation. Among the 136 discourse, we selected 68 discourses with appropriate intonation and 68 discourses with inappropriate intonation, with the same number of questions and statements in each condition. Based upon the selected 136 naturally spoken discourses, we employed Adobe Audition to create another matched 136 discourses, which reversed the final intonation, converting the appropriate to inappropriate and vice versa. Taking a selected discourse as an example, we first cut the final syllable with the opposite intonation of this selected discourse, and then spliced it with this selected discourse by replacing the final syllable of the selected discourses. This cross-splicing created another matched 136 discourses. As a result, each of the original 136 discourses has two different intonation patterns: appropriate and inappropriate. The two conditions for each discourse were lexically identical, but only differ at the final syllable.

To ensure that the two conditions of each discourse differed only in the fundamental frequency (F0) curve of the final syllables, the two final syllables for each discourse were cross-spliced in Adobe Audition to ensure their durations were identical. Moreover, Adobe Audition was also used to individually normalize the amplitude of the final syllables to ensure that the perceived loudness was equal for the two conditions. The speech materials were digitized at a sampling rate of 44.1 kHz.

A pretest for stimuli selection was conducted to avoid any difference in ecological validity between the two discourse conditions that might be caused by the cross-splicing (see pretest below). Based upon the pretest, 112 of the 136 discourses were selected and employed in the current study. Since each discourse has both an appropriate and inappropriate condition, this results in a total of 224 discourses. There were an equal number of questions and statements for the appropriate and inappropriate conditions. The two conditions were equally distributed between two lists, so that no question/answer pair was repeated within a list. Participants for each group were divided into two subgroups, with each subgroup listening to only one list of materials. In this way, all sentences were presented in both the appropriate and inappropriate format for both the amusic and control groups.

Table 2 presents some acoustic characteristics of the speech materials. Paired-sample *t*-tests showed that there was no significant difference between appropriate and inappropriate conditions for either the statements or the questions in the size of the final pitch glide or the rate of the final pitch glide (all *p*>0.05).

**Table 2:** The Mean pitch values (in semitones) for the final word of the statement and question discourses used in the appropriate and inappropriate conditions.

	Appropriate condition		Inappropriate condition		
	Statement	Question	Statement	Question	
Size of final pitch glide (st)	−2.2 (8.3)	8.3 (9.7)	−2.4 (8.6)	8.0 (8.6)	
Rate of final pitch glide (st/s)	−9.0 (33.9)	33.0 (40.2)	−12.2 (37.0)	34.1 (38.7)	
Rate (syl/s)	4.0 (0.3)		4.0 (0.3)		

### Pretest

A pretest was conducted with five native Chinese (Mandarin) speakers who did not take part in the experiments. As noted above, the pretest consisted of 136 discourses with both an appropriate and inappropriate version, resulting in a total of 272 discourses. These discourses were then divided into 4 blocks, with an equal number of the answers being spoken as a statement or as a question in each block. In addition, the two conditions of any given discourse were never presented in the same block. On two blocks the participants were required to judge if the answer sentence of the discourse was spoken as a statement using five-point Likert scale (1 = definitely not a statement to 5 = definitely is a statement). On the remaining two blocks they were to determine if the answer sentence was spoken as a question using a similar five-point Likert scale. In both cases the participants were instructed to ignore any semantic irregularities that may arise due to the answer being spoken with an inappropriate intonation. To ensure that the statement and question exemplars of all discourses used in the subsequent experiment sounded like either a statement or a question only, we averaged ratings for the statement and question exemplars of each discourse, respectively. Those discourses with mean ratings for both of the statement and question exemplars above 4 were selected for the current experiment and were used to construct 112 short discourses.

### Procedure

After the electrodes were positioned, participants were instructed to move as little as possible during the test session. A fixation cross on the computer screen was present to assist in reducing eye movements during each trial. The stimuli were presented in pseudo-random order within four blocks via loudspeakers (Microlab M-500). The participants were required to listen carefully in order to judge whether or not the discourses were semantically acceptable by pressing buttons with their forefingers of the right or the left hands after each trial. Eight practice trials were included and feedback was provided during these practice trials only.

### EEG Recording

The EEG was recorded by a NeuroScan system, with a cap containing 64 Ag/AgCl electrodes mounted according to the International 10–20 system. Vertex (Cz) served as the reference during recording, with the data subsequently re-referenced offline to the average of the left and right mastoid for analysis. The vertical eye movements and blinks were monitored via a supra- to suborbital bipolar montage. A right to left canthal bipolar montage was used to monitor for horizontal eye movements. All electrode impedances were kept below 10 kΩ during the experiment. Recording was done with a band pass filter of 0.05 Hz–100 Hz and a sampling frequency of 500 Hz.

Electro-oculogram (EOG) artifacts were automatically corrected by NeuroScan software. Data were filtered off-line with a 30 Hz low-pass filter. Critical epochs ranged from 200 ms before to 1100 ms after the acoustic onset of the critical word, with 200 ms before the onset serving as the baseline. The artifact rejection criterion was ±75 µV.

The P600 effect spans a large period of time and the time windows of 500–700 ms, 700–900 ms, and 900–1100 ms have been considered as the early, mid, and late P600 time windows (e.g., [51], [77]–[78]). Therefore, as per the literature we selected the following time windows for analysis: 100–300 ms, 300–500 ms, 500–700 ms, 700–900 ms, and 900–1100 ms. In order to compare the current results with the findings of early ERP components (N1 and N2) in [21]–[22], we further broke the 100–300 ms time windows down into four shorter time windows: 100–150 ms, 150–200 ms, 200–250 ms, and 250–300 ms.

## Results

### Behavioral Results

**Figure 1:**
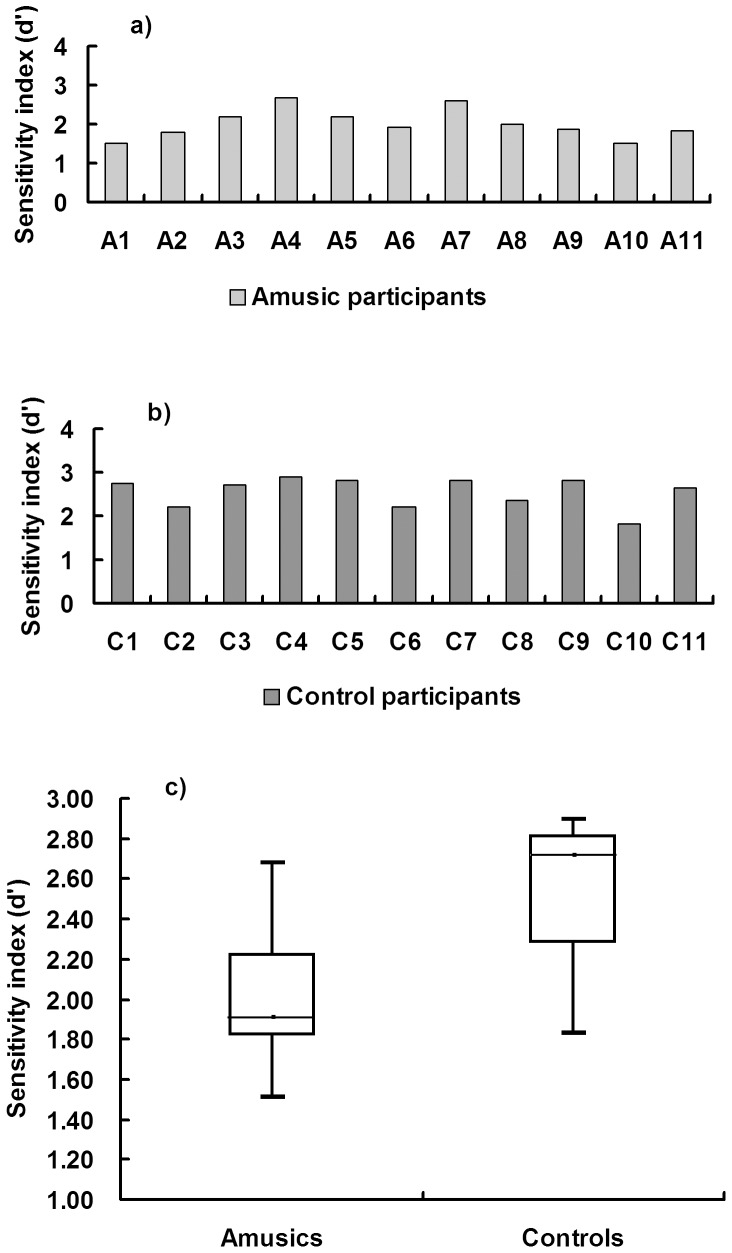
Sensitivity index (d’) for each participant in the acceptability judgment for a) amusics, b) controls, and c) box and whisker plot showing the two distributions’ minimum, 25^th^ percentile, 50^th^ percentile, 75^th^ percentile, and maximum values.

To avoid the influence of response bias, a measure of sensitivity (d’) was used to investigate the performance of two groups in judging acceptability of speech. Responding acceptable to the appropriate intonation was defined as a hit. Responding acceptable to the inappropriate intonation was defined as a false alarm. Figure 1a and 1b illustrates the performance (d’) of each participant in the acceptability judgment. Although there is some overlap between the groups (see box and whiskers plot in Figure 1c), the amusic participants as a whole do not perform as well as the controls. This was confirmed with an independent samples *t*-test revealing that there was a significant difference between the two groups [amusics mean ± SD: 2.02±3.8, controls mean ± SD: 2.55±3.5, *t* (20)  = 3.45, *p*<0.005] with the amusic group performing worse at the acceptability judgment. Moreover, individual d’ scores were significantly correlated with the individual’s melodic score from the MBEA [*r* (20)  = 0.75, *p*<0.01]. When computed within the amusic group alone, there was a significant correlation between d’ scores and the melodic score from the MBEA [*r* (9)  = 0.60, *p*  = 0.05].

### ERP Data

The EEG time window data was analyzed with the following procedure. The difference wave between the inappropriate and appropriate conditions was calculated at each electrode, including the vertical and horizontal EOG, and the mean voltage was calculated over the previously mentioned time windows. The mean voltage was then compared between groups by a *t*-test at each electrode [*t* (20) critical  =  ±2.086]. This would be similar to examining the condition by group interaction at each electrode. With 66 electrodes tested and a 5% statistical error rate means we would expect 3.3 electrodes to reach significance by chance. Chi-square was used to determine if the number of electrodes found to differ was greater than expected by statistical error (see [79] for a description of this analysis approach with large electrode montages). With 66 electrodes, a minimum of seven electrodes must show significance to exceed this criterion [χ^2^(1)  = 4.37, *p*<0.05; with only six significant electrodes, χ^2^(1)  = 2.32, *p*>0.05]. Because we tested three time windows over the P600 effect, the chi-square must be significant at *p*  = 0.05/3, or 0.0167. This equates to a minimum of eight electrodes showing a *t* value greater than the critical value. Therefore, if eight or more electrodes indicated the P600 effect, or an effect in one of the earlier time windows, differed between the groups (a condition by group interaction), then each group was analysed separately to determine how many electrodes revealed a significant inappropriate minus appropriate effect (the P600 effect within each group), again requiring eight or more electrodes to show significance before concluding the group showed a significant effect of condition. However, if no difference was found between the P600 effects (no significant condition by group interaction), then controls and amusics were combined into a single group to determine if there was an overall P600 effect (appropriate vs. inappropriate). In addition, comparisons were made after collapsing over conditions (main effect of group), but as this comparison never resulted in eight or more electrodes showing a significant difference, it is not specifically mentioned beyond this. It should be noted that the vertical and horizontal eye channels never reached significance in any of the analyses.

**Figure 2:**
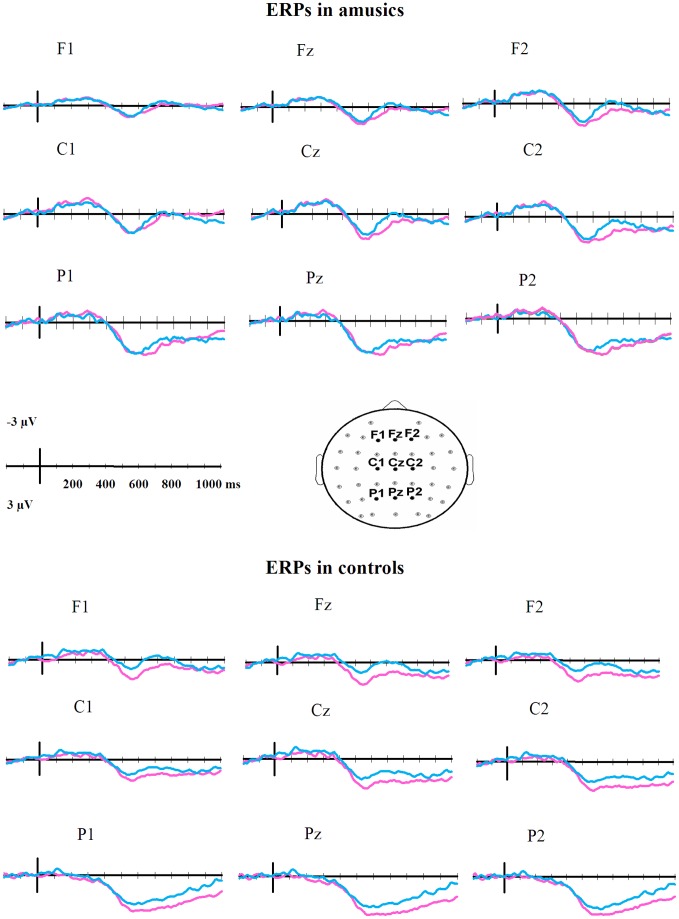
Grand-average ERPs for amusics (upper) and controls (lower) at 9 scalp sites. Blue lines show the appropriate waveform, and red lines show the inappropriate waveform. Negative is plotted up.

The appropriate and inappropriate event-related potentials for each group are shown in Figure 2 at a selection of the electrodes. The analysis of the early time windows resulted in only 3 electrodes showing a significant condition by group interaction for both the 100–300 ms time window and the 300–500 ms time window, which is not more than expected by the chance error rate [χ^2^(1) <0.03, *p*>0.05]. These time windows were then analyzed for a main effect of condition, and only one and five electrodes [both χ^2^(1) <1.67, *p*>0.05] reached significance for the 100–300 and 300–500 ms time windows, respectively.

**Figure 3:**
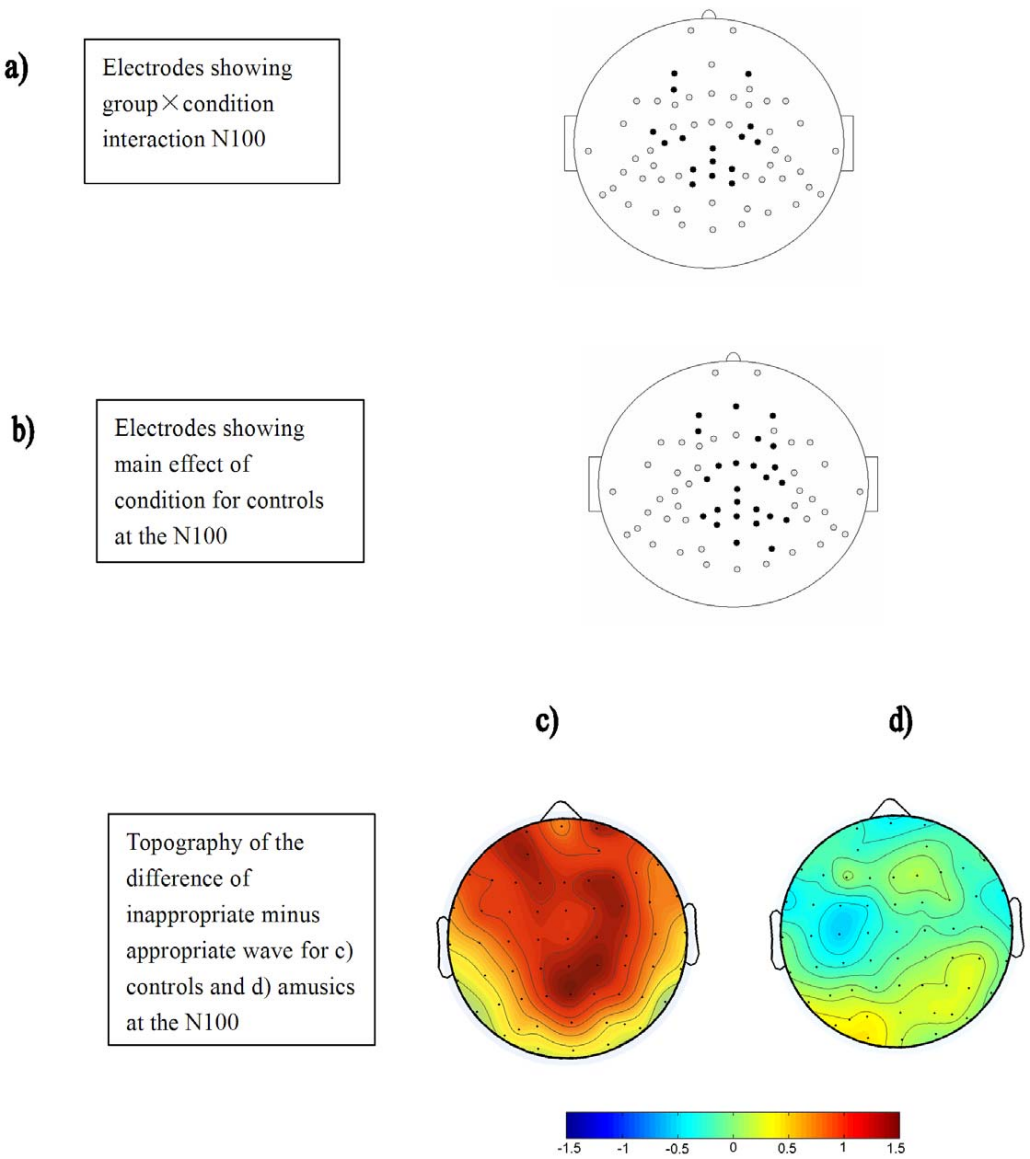
Locations of electrodes (solid black) that show a significant condition by group interaction (top row) and the effect of condition between the appropriate and inappropriate conditions for the control group (middle row) over the100–150 ms time window. The bottom row shows the voltage topography of the N100 effect for the control and amusic groups.

To further investigate whether or not there are differences in the brain activity between the controls and amusics within the early time window, the 100–300 ms time window was broken down to four 50 ms time windows: 100–150, 150–200, 200–250, and 250–300 ms. As such, the chi-square must be significant at *p*  = 0.05/4, or 0.0125 since we divided the early time window into four time windows. This also equates to a minimum of eight electrodes showing a *t* value greater than the critical value. The findings revealed that there was a significant interaction between condition and group at 100–150 ms with 16 electrodes reaching significance after a post-hoc correction [χ^2^(1)  = 51.45, *p*<0.01]. When the groups were analyzed separately, the control participants showed differences after a post-hoc correction between the inappropriate and appropriate conditions at 25 electrodes [χ^2^(1)  = 150.24, *p*<0.01] while the amusics showed differences at zero electrodes in the 100–150 ms time window. Figure 3a illustrates the electrodes that reached significance in the condition by group interaction while Figure 3b shows the electrodes that reached significance for the main effect of condition for the control group (solid black points). The finding suggests that there is no significant difference of amusics’ brain activity between the appropriate and inappropriate conditions during the 100–150 ms time window.

None of the other time windows showed a significant condition by group interaction. When combining the controls and amusics into a single group to assess the main effect of condition, none of electrodes reached significance for the 150–200 ms, and only one and five electrodes [both χ^2^(1) <1.67, *p*>0.05] reached significance for the 200–250 and 250–300 ms time windows, respectively.

**Figure 4:**
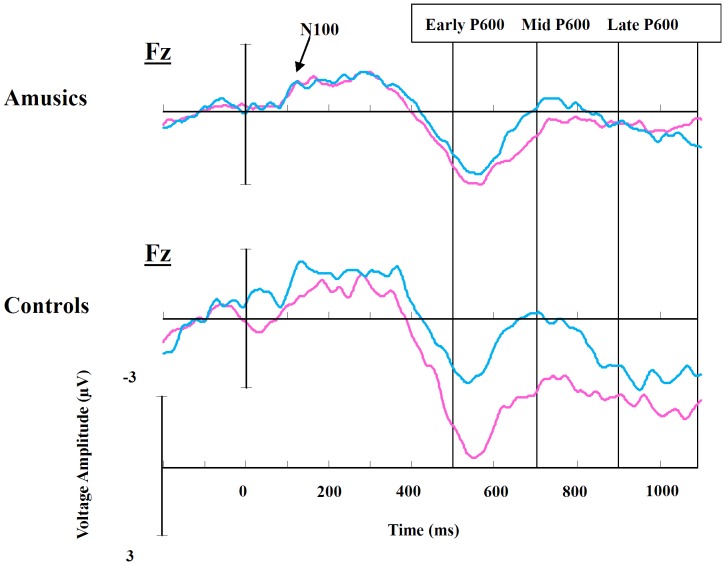
Grand-average ERPs for amusics (upper) and controls (lower) at Fz electrode. Blue lines show the appropriate waveform, and red lines show the inappropriate waveform. Negative is plotted up.

**Figure 5:**
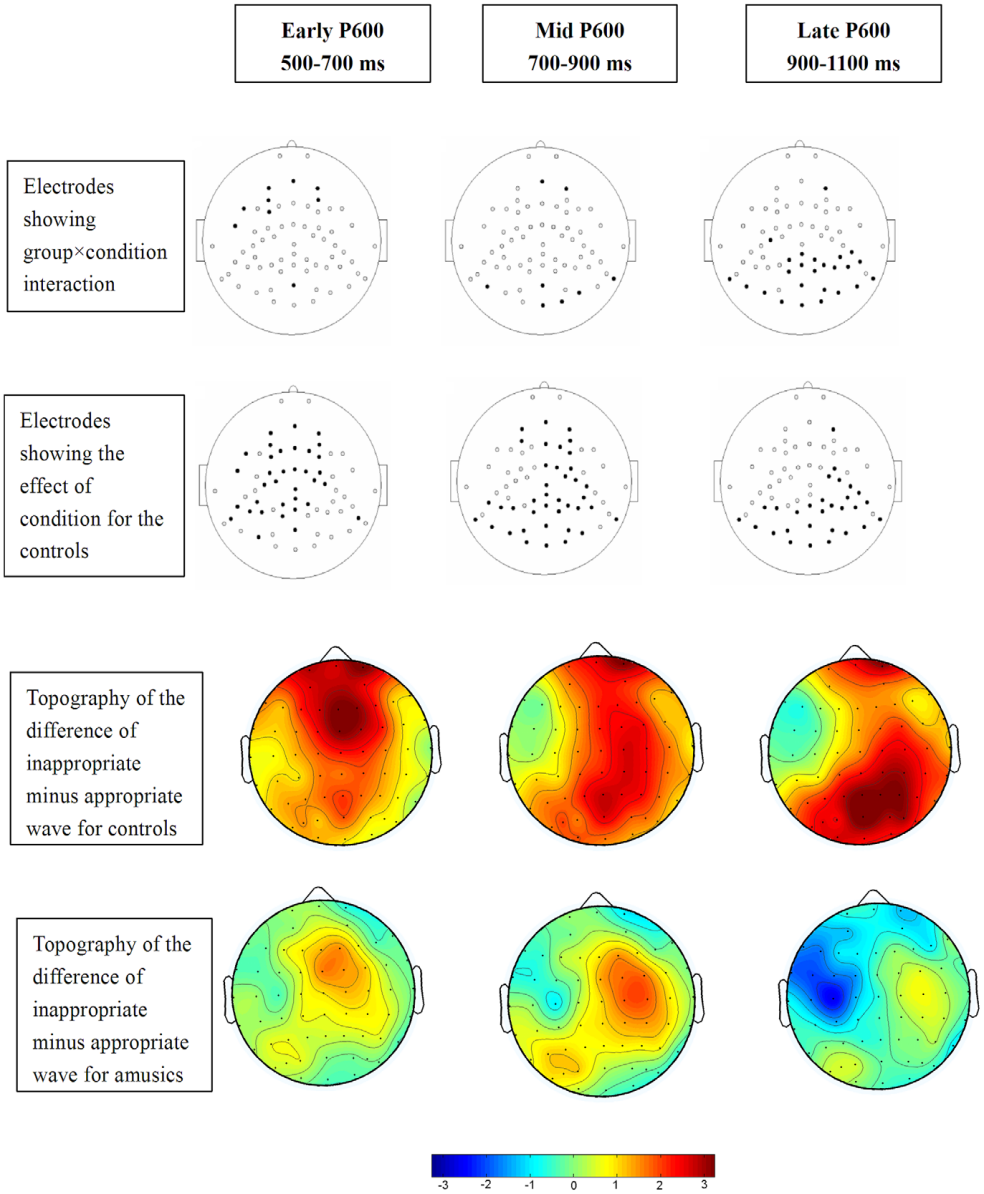
Locations of electrodes (solid black) that show a significant condition by group interaction (top row) and the effect of condition between the appropriate and inappropriate conditions for the control group (middle row) over the early (500–700 ms), mid (700–900 ms), and late (900–1100 ms) P600 time frame. The bottom row shows the voltage topography of the difference of inappropriate minus appropriate wave over the respective time windows for the control and amusic groups.

Figure 4 demonstrates the time points of interest for the P600 effect analyses: 500–700 (early P600), 700–900 (mid P600), and 900–1100 (late P600) ms time windows. The analysis revealed significant differences between the groups in the P600 effect (condition by group interaction) at all three of these time windows, with 9, 8, and 25 electrodes showing significance after a post-hoc correction at the early, mid, and late window, respectively [all χ^2^(1) >10.36, *p*s <0.02]. Separate group analysis revealed that the control participants showed differences between the inappropriate and appropriate conditions after a post-hoc correction at 35, 39, and 29 electrodes [all χ^2^(1) >210.67, *p*s <0.02] as shown in the second row of Figure 5 (solid black points), while the amusics showed differences at zero, four (C2, C4, CP2 and CP4), and three (FT7, FC5 and C3) electrodes [all χ^2^(1) <3.48, *p*s >0.05] for the early, mid, and late P600 effect time windows, respectively. The top row of Figure 5 illustrates the electrodes that reached significance in the condition by group interaction, the second row shows the electrodes that reached significance for the main effect of condition for the control group, the third row shows the voltage topography of the difference of inappropriate minus appropriate wave for the control group, and the fourth row shows the topography of the difference of inappropriate minus appropriate wave for the amusic group.

**Figure 6:**
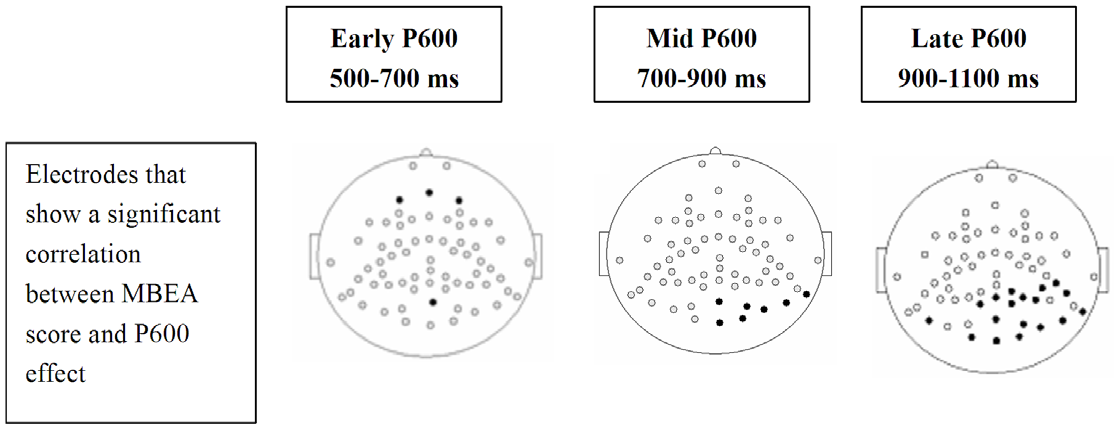
The locations of the electrodes (solid black) that show a significant correlation between participants’ melodic scores from the MBEA and their P600 effects.

The individual mean voltage difference over each time window was correlated with an individual’s melodic score from the MBEA. This resulted in 4, 7, and 18 electrodes showing P600 effects that correlated with the individual’s melodic score from the MBEA for the early, mid, and late P600 time windows, respectively [all *r* (20) >0.42, *p*s <0.05]. These electrodes are shown in Figure 6. However, when computed within the amusic group alone, none of electrodes showed a significant correlation between the melodic MBEA score and the P600 effect at any of the time windows (*p*s >0.05). The amusics’ melodic scores from the MBEA were correlated with their voltage amplitudes of the appropriate condition at 1, 21, and 13 electrodes in the early, mid, and late P600 time windows [all *r* (9) >0.60, *p*s <0.05].

## Discussion and Conclusion

The main question under investigation in the current study was whether or not Mandarin speaking individuals with amusia show deficits in the processing of intonation in prosody during speech comprehension. The behavioral data indicate that the classifying of prosody as appropriate or inappropriate is impaired in amusic individuals. In addition, we present the first electrophysiological evidence that supports the behavioral findings, and revealed that the control participants showed a larger P600 effect and a smaller N100 effect in response to inappropriate relative to appropriate prosody. In contrast, the brain activities of the amusic participants did not significantly differ between the appropriate and inappropriate conditions. Therefore, both the behavioural and the electrophysiological measures indicate that the amusics are impaired, relative to controls, in the processing of intonation in prosody during speech comprehension.

Although a slight difference in N100 generator loci between the amusic and control groups has been reported previously, these authors suggested that the N100 component appeared normal in amusia [21]. In contrast to this result, the current data did not show a significant difference in amplitudes between the inappropriate and appropriate conditions in N100 component for the amusic participants. The discrepancy between this study and the previous one may be attributed to the different experimental designs, stimulus types, and methods. The previous study [21] used the oddball paradigm with tones to investigate the performance of pitch change detection in amusia, while the current study focused on the speech comprehension in amusia by testing them with an acceptability judgment of speech prosody.

Indeed, the N100 can reflect sudden changes in sound energy, such as acoustic changes [80]. Increased N100 amplitudes can be also generated when the listener attends to relevant stimuli, while the small N100 occurs when ignoring unpredictable irrelevant stimuli [81]. In the current study, the syntactic and semantic context within the discourses allows the listeners to create a strong intonation expectation. For the controls, this expectation elicits a larger N100 when the intonation is congruent than when it is incongruent. The lack of differentiation in the N100 effect between the inappropriate and appropriate conditions for the amusics may be attributed to the failure to reinterpret as stated above. Moreover, this is also consistent with the evidence by previous studies suggesting that amusics have difficulties in discriminating the different pitch contours [6], [10] underlying the prosody of speech.

Although the most common view is that the P600 reflects the processing of syntactic violations that produce grammatical errors [51]–[53], a mismatch between syntax and prosody also elicits a P600 effect [57]–[58], as noted above. Similar to western language speakers, the Mandarin-speaking controls in the current study showed a large P600 effect when presented with a mismatch between syntax and prosody in Mandarin. In contrast, the Mandarin-speaking amusics did not show a significant difference between the appropriate and inappropriate conditions in the P600 component. This may be due to that, when the incongruent intonation in the final syllable is observed, the controls may track back to the beginning of the answer sentence in an attempt to make sense of the unexpected question/statement construction, while the amusic participants may fail to process the violations of the constraints created by long-distance dependencies due to their deficits in short-term and working memory [13]–[15]. Moreover, the distribution of the P600 effect for the control participants is initially frontal, and then shifts to a posterior maximum. This fits with the notion of an initial revision attempt [59], which ultimately ends in syntactic failure due to processing difficulty [51], [70]. The lack of a significant early P600 effect over the frontal region in amusia is in line with previous research demonstrating that amusic individuals with non-tonal language fail to exhibit a P600 effect in judging anomalous notes in a musical context [22].

The current results are in line with behavioral studies demonstrating that amusics have deficits in intonation processing [10], [32]–[35] and extend this to suggest that pitch deficits in speech perception have affected speech comprehension for Mandarin amusics. Moreover, the results of the current study are in contrast to previous work where Mandarin amusics showed normal intonation processing in sentences ranging from three to seven syllables [37]. One possible explanation may be the differences in demand of memory loads between the two studies. Compared to the relatively short speech materials previously used [37], in the current study the short discourses ranged from 15 to 27 syllables. In addition the participants were required to make a semantic acceptability judgment. Although the participants might be aware that pitch changes of intonation would occur at the end of the discourse, the semantic acceptability judgment requires careful listening to the whole discourse. This comprehension requirement results in an increased memory load (e. g. [82]–[85]). Therefore, the current speech comprehension task may place more of a burden on memory resources in storing linguistic information for analysis than that of the previous study [37]. A second possible explanation may be the difference in the difficulty of integration of prosody and context between the two studies. In order to make an acceptability judgment in the current study the participants would have to integrate the intonation into the context of the discourse. The greater the mismatch the greater the difficulty they would have to successfully integrate the prosody with the context [83]. Therefore, the semantic acceptability judgment requires more complicated processing than the discrimination and identification of prosody at the perceptual level [37].

The current data demonstrate the importance of using objective measures rather than relying on self report in order to detect subtle deficits that may go unnoticed in the day to day use of language. Two factors may account for this difference in findings between the objective measures and the subjective reports. Generally, people use appropriate intonation and rarely speak with inappropriate prosody during daily communication. Combined with the fact that the behavioral data do indicate that the amusic participants can perform the discrimination (d’ all above 1), it may be that they simply are never in a situation where they could be expected to experience a negative influence of their pitch deficits on speech comprehension. Furthermore, it has been suggested that some cues (syntactic, semantic, and contextual) of language [24], combined with additional non-pitch-based cues (duration and intensity) in speech [37] may provide sufficient information for understanding speech. Semantic constraints have been shown to reduce the P600 effects under investigation in some circumstances [59]. From this perspective, even though the objective measures show that the amusic individuals are less sensitive to speech prosody, the above cues may more than adequately compensate speech comprehension during daily communication for amusics.

In conclusion, the current study shows that amusic individuals whose first language is Mandarin do have problems in classifying prosody as appropriate or inappropriate, as indexed by the lower d’ measures. In addition, the amusic participants did not show a significant difference between appropriate and inappropriate conditions in either their N100 or the P600. In contrast, the controls showed a reduced N100 in response to inappropriate prosody, and elicited the expected P600 effect. This suggests that the pitch processing deficit of amusia may also affect speech comprehension, and supports the resource-sharing framework suggesting that language and music may share some cognitive and neural resources [23]–[25].
